# Unraveling the Relationship Between Osteoporosis, Treatment Modalities, and Oral Health: A Comprehensive Review

**DOI:** 10.7759/cureus.49399

**Published:** 2023-11-25

**Authors:** Nandini Sharma, Amit Reche

**Affiliations:** 1 Dentistry, Sharad Pawar Dental College, Datta Meghe Institute of Higher Education and Research, Wardha, IND; 2 Public Health Dentistry, Sharad Pawar Dental College, Datta Meghe Institute of Higher Education and Research, Wardha, IND

**Keywords:** dental procedures, multidisciplinary approach, bisphosphonates, treatment modalities, oral health, osteoporosis

## Abstract

This review delves into the intricate interplay between osteoporosis, its treatment approaches, and oral health. The examination underscores the substantial impact of osteoporosis, characterized by reduced bone density, on various oral health parameters such as periodontal health, tooth loss, and jawbone density. While pharmacological interventions, including bisphosphonates and hormone replacement therapy, play a crucial role in managing osteoporosis, they necessitate careful consideration, particularly about the risk of osteonecrosis of the jaw. A comprehensive approach involving collaboration between dentists and healthcare providers is imperative for holistic patient care. Implementing screening protocols for osteoporosis in dental settings and meticulously planning dental procedures for patients undergoing osteoporosis treatments are vital aspects of clinical practice. This review also sheds light on emerging trends in osteoporosis research, such as the influence of genetic factors and the microbiome, emphasizing the necessity for innovative treatment modalities. In conclusion, the review provides valuable insights into the nuanced connections between osteoporosis and oral health, thereby laying a foundation for informed clinical practices and guiding future research initiatives. Furthermore, it highlights the importance of optimizing dental procedures and assessing long-term oral health outcomes as critical avenues for future research endeavors.

## Introduction and background

Osteoporosis, a prevalent systemic skeletal disorder characterized by reduced bone density and structural deterioration, represents a significant global public health challenge. This condition leads to porous and fragile bones, increasing the risk of fractures, especially in weight-bearing areas such as the spine, hip, and wrist [1]. While the characteristics of osteoporosis have been elucidated in the opening paragraph, an alternative connector is required to enhance coherence. Beginning with a systematic introduction is crucial for better reader comprehension. Embracing the funnel approach, we initiate by presenting the broader context of the topic and progressively narrowing down the focus to provide more specific details and insights, guiding readers through the content.

Osteoporosis involves a systemic weakening of bone strength, resulting in an elevated susceptibility to fractures. Often asymptomatic until a fracture occurs, early detection and intervention are paramount for effective management of this condition. Key assessments, such as dual-energy X-ray absorptiometry (DXA), are pivotal in diagnosing osteoporosis and evaluating fracture risk [2]. Understanding the intricate relationship between osteoporosis, its treatment, and oral health is paramount. As a systemic disorder, osteoporosis has implications beyond the skeletal system, impacting various aspects of health. Recent research has highlighted connections between osteoporosis and oral health parameters, including periodontal health, tooth loss, and jawbone density. Additionally, the influence of osteoporosis treatment modalities on oral health introduces a new dimension to patient care, necessitating a holistic approach [3].

This review seeks to consolidate knowledge about the multifaceted interactions between osteoporosis, its treatment modalities, and oral health. By synthesizing existing literature, we aim to provide a comprehensive understanding of how osteoporosis influences oral health and how osteoporosis treatments may impact dental outcomes. Furthermore, we aim to explore the potential for an interdisciplinary approach to patient care, fostering collaboration between healthcare providers and dental professionals.

## Review

Osteoporosis: a systemic bone disorder

Definition and Characteristics of Osteoporosis

Osteoporosis is a systemic bone disorder characterized by low bone mass and structural deterioration of bone tissue, leading to increased bone fragility and susceptibility to fractures. The hallmark of osteoporosis is reduced bone mineral density (BMD), resulting in porous and weakened bones. Microscopically, there is a disruption in the trabecular bone architecture, compromising its strength and integrity. This diminished bone quality distinguishes osteoporotic bones from healthy ones and underscores the clinical significance of the condition [4]. Beyond the microstructural changes, osteoporosis is often considered a silent disease, as it may progress without overt symptoms until a fracture occurs. Fractures associated with osteoporosis commonly involve the spine, hip, and wrist, significantly impacting an individual's quality of life and independence [5].

Prevalence and Demographics

The prevalence of osteoporosis varies across populations and is influenced by age, sex, race, and geographic location. According to a systematic review and meta-analysis, the global prevalence of osteoporosis and osteopenia was estimated to be 19.7% and 40.4%, respectively, with significant variation between countries and regions [6]. In the United States, the prevalence of osteoporosis among men and women over 50 years of age is reported to be 4.2% and 18.8%, respectively [7]. Worldwide, osteoporosis is estimated to affect 200 million women, approximately one-tenth of women aged 60, one-fifth of women aged 70, two-fifths of women aged 80, and two-thirds of women aged 90 [8]. Additionally, osteoporosis is a disease that affects people of all races/ethnicities and both sexes, with an estimated 10 million people aged 50 years and older in the United States having osteoporosis, the majority of whom are women [9].

Demographic factors, such as ethnicity and geographic location, play a significant role in influencing the prevalence of osteoporosis. Certain populations demonstrate a heightened susceptibility, and it is essential to delve into specific regional differences that may arise from genetic, environmental, and lifestyle factors. A nuanced understanding of these demographic variations is imperative to enhance the effectiveness of preventive strategies and healthcare interventions. This knowledge allows for the tailoring of interventions to address the unique needs of specific at-risk groups [10].

Risk Factors and Etiology

Several risk factors contribute to osteoporosis development, including genetic, hormonal, lifestyle, and medical factors. A family history of osteoporosis or fractures, low body weight, and a history of fractures in adulthood are among the genetic and familial risk factors. Hormonal factors, particularly the decline in estrogen levels in postmenopausal women, contribute significantly to bone loss. Lifestyle factors such as sedentary behavior, smoking, excessive alcohol consumption, and a diet deficient in calcium and vitamin D are recognized as modifiable risk factors [11].

Medical conditions and medications also play a role in osteoporosis etiology. Chronic diseases like rheumatoid arthritis, inflammatory bowel disease, and endocrine disorders can impact bone health. Furthermore, certain medications, including glucocorticoids and some anticonvulsants, are associated with an increased risk of osteoporosis [12]. Understanding osteoporosis's multifaceted etiology and risk factors is essential for implementing targeted prevention and intervention strategies and informing the comprehensive management of the condition [13].

Oral health and osteoporosis

The Connection Between Oral Health and Systemic Conditions

Oral health is intricately connected to overall systemic well-being, as evidenced by a growing body of research establishing links between oral health and various systemic conditions. The oral cavity serves as a window to systemic health, with manifestations of certain diseases appearing in the mouth. This interconnection is exemplified by bidirectional relationships, where systemic conditions may influence oral health and vice versa. Understanding this dynamic relationship is crucial for comprehensive patient care and underscores the importance of considering oral health as an integral component of overall health [14].

Impact of Osteoporosis on Oral Health

Periodontal health: Osteoporosis can significantly impact periodontal health, influencing the supporting structures of the teeth. Research suggests that individuals with osteoporosis may exhibit an increased susceptibility to periodontal disease, characterized by inflammation of the gums and progressive destruction of the alveolar bone. The compromised bone density in osteoporosis can contribute to weakened periodontal support, potentially exacerbating periodontal conditions. Conversely, periodontal disease may contribute to systemic inflammation, potentially influencing bone metabolism and osteoporosis. This complex interplay necessitates a holistic approach to managing osteoporosis and periodontal health [15].

Tooth loss: Osteoporosis has been associated with an elevated risk of tooth loss, potentially stemming from compromised bone density and changes in the oral environment. Reduced bone mass in the jaw may affect the stability and support of teeth, leading to an increased likelihood of tooth mobility and subsequent loss. Understanding this association is crucial for preventive dental care strategies, as preserving oral health becomes integral to minimizing tooth loss risk in osteoporosis individuals [16].

Bone density in the jaw: The jawbone, a critical component of the oral anatomy, can be influenced by systemic osteoporosis-related changes. Diminished bone density in the jaw may contribute to dental procedure challenges, such as implant placement and oral surgery. This has implications for dental practitioners, necessitating considerations of bone health and potential adaptations in treatment planning for individuals with osteoporosis [17].

Treatment modalities for osteoporosis

Pharmacological Interventions

Bisphosphonates: Bisphosphonates are among the most prescribed osteoporosis medications- alendronate and risedronate work by inhibiting bone resorption, primarily by osteoclasts. By slowing down the breakdown of bone tissue, bisphosphonates help to maintain or increase bone density, reducing the risk of fractures. However, long-term use has been associated with potential side effects, notably osteonecrosis of the jaw (ONJ) and atypical femoral fractures. Regular monitoring and careful consideration of the duration of treatment are essential, particularly in specific populations, to manage potential risks [18].

Hormone replacement therapy (HRT): Hormone replacement therapy involves the use of hormones, often estrogen, to address hormonal deficiencies, particularly in postmenopausal women. Estrogen plays a crucial role in maintaining bone density, and HRT aims to counteract the bone loss associated with hormonal changes. While effective in reducing fracture risk, HRT is subject to ongoing research and individualized risk-benefit assessments. Concerns about potential risks, including cardiovascular complications and an increased risk of certain cancers, have led to a more cautious approach to prescribing HRT. Decisions about its use should be made on a case-by-case basis, considering individual health profiles and risks [19].

Selective estrogen receptor modulators (SERMs): SERMs, such as raloxifene, act as estrogen agonists in some tissues and antagonists in others. In the context of osteoporosis, SERMs positively affect bone density without the same level of risk as systemic estrogen therapy. Raloxifene, for example, is often considered in postmenopausal women who may not be suitable candidates for traditional hormone replacement therapy. SERMs provide an alternative option for managing osteoporosis in specific populations, with a more favorable risk profile compared to systemic estrogen therapy [20].

Denosumab: Denosumab is a monoclonal antibody that targets a receptor called RANKL (receptor activator of nuclear factor kappa-B ligand), which is involved in osteoclast function. By inhibiting RANKL, denosumab reduces bone resorption and enhances bone density. It is administered subcutaneously and has demonstrated efficacy in reducing fracture risk. Regular monitoring is essential with denosumab due to the potential for rebound bone loss upon discontinuation. This medication is often considered for individuals who may not tolerate or respond well to other osteoporosis treatments [21].

Non-pharmacological Approaches

Lifestyle modifications: Lifestyle changes play a significant role in managing osteoporosis, particularly in mitigating risk factors associated with bone loss. Smoking cessation is crucial because smoking has been linked to decreased bone density and an increased risk of fractures. Similarly, excessive alcohol consumption has been associated with an elevated risk of osteoporosis and fractures. Modifying these behaviors positively influences bone health and reduces the likelihood of fractures. Healthcare professionals often emphasize the importance of lifestyle modifications in osteoporosis management [22].

Dietary considerations: Adequate nutrition is essential for optimal bone health. Calcium and vitamin D are particularly critical, as calcium is a crucial component of bone structure and vitamin D aids absorption. Dietary sources rich in calcium include dairy products, leafy green vegetables, and fortified foods. The skin synthesizes vitamin D in response to sunlight, and dietary sources include fatty fish and fortified products. In cases where dietary intake may be insufficient, supplements may be recommended. Ensuring an appropriate balance of these nutrients is fundamental for supporting bone health and preventing deficiencies that could contribute to osteoporosis [23].

Exercise and physical activity: Regular exercise, especially weight-bearing and resistance training, is a cornerstone of osteoporosis management. Weight-bearing exercises like walking, jogging, or dancing stimulate bone formation and help maintain bone density. Resistance training, including activities like weightlifting, contributes to overall bone health by exerting stress on bones and promoting bone strength. Considering age and overall health, exercise regimens should be tailored to an individual's capabilities and needs. Various exercises that focus on different muscle groups and bone-loading activities are recommended. Physical activity supports bone health and contributes to overall well-being and balance, reducing the risk of falls and fractures [24].

Oral health implications of osteoporosis treatment

Effects of Bisphosphonates on Oral Health

Osteonecrosis of the jaw (ONJ): One of the most noteworthy oral health concerns associated with bisphosphonate therapy is the development of osteonecrosis of the jaw (ONJ). ONJ is a rare but severe condition characterized by the death of jawbone tissue, often following dental procedures or trauma. The precise mechanisms underlying ONJ are not fully elucidated, but bisphosphonates' prolonged suppression of bone turnover may contribute to impaired healing. Dental professionals must exercise caution when considering invasive dental procedures for patients on long-term bisphosphonate therapy. Preventative measures such as good oral hygiene and regular dental check-ups are essential for monitoring and minimizing the risk of ONJ [25].

Dental extractions and precautions: Dental extractions in individuals undergoing bisphosphonate treatment pose a unique challenge due to the increased risk of ONJ. Dental practitioners often consider a conservative approach, avoiding unnecessary extractions and opting for alternative treatments when possible. Precautionary measures, such as a thorough assessment of the patient's medical history and collaboration between the treating physician and dentist, are crucial in minimizing the risk of ONJ associated with dental extractions [26].

Other Treatment Modalities and Their Impact on Oral Health

Hormone replacement therapy (HRT): Hormone replacement therapy can influence oral health, particularly in postmenopausal women. Estrogen, a key component of HRT, contributes to maintaining oral tissues, including the gums and salivary glands. HRT may alleviate some oral symptoms associated with menopause, such as dry mouth and an increased risk of periodontal disease. However, the use of HRT is subject to individualized risk-benefit assessments, and its impact on oral health must be considered in the broader context of systemic effects [27].

Denosumab and its effects on bone metabolism: Denosumab, a monoclonal antibody targeting RANKL, has implications for bone metabolism, including the jawbone. While denosumab has shown efficacy in reducing fracture risk, its effects on oral health are less extensively studied than bisphosphonates. Dental practitioners should be aware of potential impacts on bone healing and collaborate closely with physicians to manage patients receiving denosumab, particularly in dental surgeries or extractions [28].

Interdisciplinary approach to patient care

Collaboration Between Dentists and Healthcare Providers

Effective patient care in osteoporosis demands a collaborative effort between dentists and healthcare providers from various disciplines. This collaboration is paramount for ensuring comprehensive, integrated care that addresses skeletal and oral health [29]. Dentists, as primary oral healthcare providers, should actively communicate with physicians, endocrinologists, and other relevant healthcare professionals involved in the management of osteoporosis. Sharing patient medical histories, treatment plans, and monitoring protocols enables a cohesive approach to address the potential oral health implications of osteoporosis and its treatments. This collaboration is crucial when considering dental procedures impacting bone health, such as extractions or implant placements [30].

Screening Protocols for Osteoporosis in Dental Settings

Dental settings offer a unique opportunity to identify individuals at risk of osteoporosis early. Integrating screening protocols for osteoporosis within routine dental assessments can enhance the identification of at-risk patients and facilitate timely referral for further bone density evaluation [31]. Dental professionals can implement tools such as questionnaires, clinical risk assessment, and dental imaging (e.g., panoramic radiographs) to identify potential indicators of osteoporosis. Positive findings should prompt discussions with patients about their bone health and referral to healthcare providers for comprehensive evaluation and bone density testing when appropriate. This collaborative screening approach fosters a proactive stance in managing osteoporosis and its potential impact on oral health [32].

Communication Between Healthcare Professionals for Holistic Patient Care

Effective and transparent communication among healthcare professionals ensures comprehensive patient care. When managing individuals with osteoporosis, dentists, physicians, and other specialists must collaborate and exchange pertinent information, fostering the development of a unified and patient-centered management plan [33]. This communication should occur regularly, encompassing updates on medication changes, treatment plan adjustments, and any oral health concerns identified during dental visits. Additionally, dental professionals should proactively communicate information about upcoming dental procedures that may pose a risk, facilitating collaborative decision-making and the implementation of preventive measures [34]. Moreover, conducting interdisciplinary case conferences or meetings provides a valuable platform for in-depth discussions. These forums enable healthcare teams to collectively address the complexities of patient care, share insights, and formulate collaborative strategies. This collaborative approach ensures that the management of osteoporosis aligns with broader healthcare objectives, underscoring the significance of oral health in the patient's overall well-being [35].

Future directions and research needs

Emerging Trends in Osteoporosis Research

Genetic factors and precision medicine: Investigating the genetic factors underlying osteoporosis is essential for developing more personalized and effective interventions. Osteoporosis has a hereditary component, and understanding the genetic basis can help identify individuals at higher risk. Moreover, researching individual variations in treatment response can pave the way for precision medicine approaches. This involves tailoring treatments based on an individual's genetic profile optimizing therapeutic outcomes while minimizing potential side effects. Identifying specific genetic markers associated with osteoporosis risk and treatment responsiveness can enhance early detection, prevention, and management strategies, ushering in a new era of precision medicine in bone health [36]. Several research studies have been conducted on the connection between oral health and systemic conditions. Periodontal disease has been associated with a number of health conditions, including heart disease, diabetes, metabolic syndrome, obesity, eating disorders, liver disease, and pregnancy-related complications [37-39]. While many associations have been found between periodontitis and systemic conditions, finding direct causality remains elusive [38-39]. The negative impact of oral infection on systemic health generally stems from the entry of oral microorganisms or their products into the bloodstream, which is known as bacteremia [40]. Signs of several systemic diseases and conditions can be manifested in the mouth, which makes the oral cavity an important diagnostic tool for early detection and referral to the appropriate health professional [40]. Therefore, it is important to educate oneself on the relationship between oral health and systemic disease, as systemic disease continues to rise and detection and prevention are more important now than ever before [41].

Microbiome and bone health: The relationship between the gut microbiome and bone metabolism is an evolving field with significant implications. Current research indicates that the composition of the gut microbiota plays a pivotal role in influencing the absorption of essential nutrients crucial for bone health, including calcium and vitamin D. Moreover, there is mounting evidence pointing towards a correlation between the gut microbiome and the onset of osteoporosis. It is imperative to specify the microorganisms involved in these interactions and provide a justification for the interconnectedness observed. This nuanced understanding could potentially unveil novel therapeutic strategies. Exploring the manipulation of the gut microbiome through interventions such as probiotics or dietary modifications may emerge as a complementary approach to traditional osteoporosis treatments. By specifying the microorganisms involved and justifying the interconnected nature of these relationships, this avenue of research holds the promise of uncovering innovative ways to preserve and improve bone health, considering the integral role of the microbiome in overall bone metabolism [42].

Biomarkers for fracture risk assessment are indispensable in refining risk stratification for individuals with osteoporosis. For readers unfamiliar with the term, biomarkers are measurable indicators reflecting physiological or pathological processes within the body. In osteoporosis research, identifying and validating specific biomarkers is an active area of investigation. These biomarkers offer nuanced insights into various facets of bone health, including turnover, density, and quality. Examples of biomarkers include bone turnover markers (BTMs), such as serum C-terminal telopeptide of type I collagen (CTX) and serum N-terminal propeptide of type I collagen (P1NP). BTMs reflect the rate of bone remodeling, providing information about bone formation and resorption. High levels of CTX and P1NP may indicate increased bone turnover, suggesting an elevated risk of fractures. Additionally, markers like serum 25-hydroxyvitamin D (25(OH)D) indicate vitamin D status, which is crucial for bone health. The significance of these biomarkers lies in their potential to serve as precise indicators of an individual's fracture risk. By measuring and interpreting these biomarkers, clinicians can tailor interventions more effectively. For instance, increased attention to individuals with elevated BTMs or low vitamin D levels may be warranted, allowing for targeted preventive measures. Accurate risk assessment, facilitated by biomarkers, is paramount for tailoring interventions to those most in need. The ongoing exploration of specific biomarkers in osteoporosis research underscores a commitment to advancing the field, aiming to develop more sophisticated and individualized approaches to fracture prevention and management [43].

Potential Innovations in Treatment Modalities

Biologics and targeted therapies: Exploring and developing biologic agents and therapies represent a promising frontier in osteoporosis treatment. Biologics are substances derived from living organisms that can precisely modulate biological processes. In osteoporosis, these agents target and influence critical pathways involved in bone metabolism. Advancements in our understanding of molecular mechanisms related to bone remodeling have paved the way for the creation of therapies that can selectively target specific cellular pathways. This targeted approach holds the potential for more personalized and effective treatments, allowing clinicians to address the root causes of bone loss with greater precision [44].

Drug delivery systems: Innovations in drug delivery systems can enhance osteoporosis medication safety and effectiveness. Controlled-release formulations and alternative administration routes are areas of active research. Controlled-release formulations can sustain medication release over time, improving patient adherence by reducing dosing frequency. Alternative administration routes, such as transdermal patches or injections, offer new options for patients who may experience side effects with traditional oral medications. These advancements aim to improve the overall patient experience and mitigate potential side effects associated with the long-term use of certain osteoporosis medications, such as bisphosphonates [45].

Combination therapies: Investigating the potential synergies of combining different classes of osteoporosis medications is an essential avenue for research. Combination therapies involve using drugs from different classes to address multiple aspects of bone metabolism. This approach recognizes that bone health is a complex interplay of various factors, and a combination of medications may provide a more comprehensive and effective treatment strategy. By targeting different pathways or mechanisms simultaneously, combination therapies have the potential to achieve greater efficacy and address the multifaceted nature of osteoporosis. This area of exploration is vital for optimizing treatment outcomes and providing patients with more robust solutions for managing their bone health [46].

Areas for Further Investigation in the Context of Oral Health

Impact of new treatment modalities on oral health: As advancements in osteoporosis treatments continue, it becomes imperative to delve into how these novel therapies affect oral health. This involves researching the specific repercussions of medications such as monoclonal antibodies and targeted therapies on oral tissues and bone metabolism. Since oral health is intricately linked to overall well-being, comprehending the potential side effects or benefits of these emerging treatments on the oral cavity is crucial. This knowledge can guide dentists and healthcare professionals in tailoring dental care plans for patients undergoing these newer forms of osteoporosis treatment [47].

Optimizing dental procedures for osteoporotic patients: Individuals with osteoporosis may face unique challenges when undergoing dental procedures like extractions or implant placements. Research should aim to identify and optimize protocols that address the specific needs of this patient population. This includes developing strategies to minimize the risk of jaw osteonecrosis, a rare but severe condition associated with certain osteoporosis medications. Additionally, enhancing postoperative healing processes in osteoporotic patients is a crucial consideration. Optimizing dental procedures for this demographic ensures safer and more effective interventions, improving oral health outcomes [48].

Long-term oral health outcomes of osteoporosis treatment: Longitudinal studies are essential to assess the enduring effects of prolonged osteoporosis treatment on oral health. This involves tracking patients over extended periods to evaluate the cumulative impact of medications such as bisphosphonates, denosumab, and other emerging therapies. Researchers should focus on periodontal health, tooth retention, and jawbone density parameters. Understanding the long-term consequences of osteoporosis treatments is vital for clinicians to make informed decisions about patient care and for patients to comprehend potential oral health implications associated with these medications. It contributes to a more comprehensive understanding of the overall health outcomes for individuals undergoing extended osteoporosis treatment [49].

## Conclusions

This comprehensive review delved into the intricate connections between osteoporosis, its treatment modalities, and oral health, revealing a dynamic interplay extending beyond skeletal health's boundaries. The summary of key findings underscores the far-reaching implications of osteoporosis on oral health, encompassing periodontal health, tooth loss, and jawbone density. Pharmacological interventions, including bisphosphonates and hormone replacement therapy, carry significant implications for oral health, emphasizing careful consideration in dental practice. The multifaceted nature of this relationship emphasizes the importance of a multidisciplinary approach to patient care, necessitating collaboration between dentists and healthcare providers. This collaborative model is crucial for navigating the complexities of treatment planning and preventive strategies. In clinical practice, dentists should remain vigilant in assessing oral health in individuals with osteoporosis, while screening protocols in dental settings could enhance the early identification of at-risk patients. Future research directions include exploring emerging trends in osteoporosis research, such as genetic factors and the microbiome, and investigating potential innovations in treatment modalities. Optimizing dental procedures for individuals with osteoporosis and assessing long-term oral health outcomes require further investigation. This review provides a foundation for informed clinical practices and future research endeavors, contributing to a more holistic approach to managing osteoporosis and its intricate relationship with oral health.
